# Paeonol Alleviate Cisplatin‐ and Noise‐Induced Hearing Loss by Preserving Hair Cell Ribosome Biogenesis

**DOI:** 10.1002/cns.71115

**Published:** 2026-09-03

**Authors:** Minhui Xia, Tianshi Sun, Wenyu Hou, Yiyun Lou, Yunjie Li, Hua Jiang, Hui Chai, Jiaoyao Ma, Mingyu Xia

**Affiliations:** ^1^ School of Life Sciences Zhejiang Chinese Medical University Hangzhou China; ^2^ State Key Laboratory of Medical Neurobiology, Otorhinolaryngology Department of Eye and ENT Hospital, ENT Institute, MOE Frontiers Center for Brain Science Fudan University Shanghai China; ^3^ NHC Key Laboratory of Hearing Medicine Fudan University Shanghai China; ^4^ Department of Otolaryngology The Second Affiliated Hospital Zhejiang University School of Medicine Hangzhou China

**Keywords:** hair cell, hearing loss, otoprotection, paeonol

## Abstract

**Background:**

Hearing loss primarily results from irreversible cochlear hair cell (HC) damage caused by factors such as noise, aging, genetics, and ototoxic drugs including cisplatin. Currently, there are no effective clinical otoprotective agents available. The natural compound paeonol, derived from Paeonia suffruticosa, exhibits potential anti‐inflammatory and antioxidant properties, positioning it as a promising therapeutic candidate given the central roles of oxidative stress and inflammation in hearing loss pathogenesis.

**Methods and Results:**

Using mouse cochlear explant cultures, we demonstrated that paeonol protects against cisplatin‐induced HC damage in a concentration‐dependent manner and reduces the accumulation of MitoSOX, TNF‐α, and IL‐6. In vivo, paeonol administration not only prevented cisplatin‐induced HC loss and inner HC synaptic degeneration but also conferred protection against noise‐induced HC damage and hearing loss, demonstrating broad otoprotective efficacy. Mechanistically, RNA‐seq analysis revealed that paeonol exerts its protective effect partly by preventing ribosome biogenesis dysfunction, thereby ensuring normal protein synthesis in HCs.

**Conclusions:**

Together, our work demonstrates the efficacy of paeonol across multiple models and reveals a potential protective mechanism, paving the way for its development as a much‐needed otoprotective drug.

## Graphical abstract

Paeonol protects against cisplatin‐ and noise‐induced hearing loss by mitigating oxidative damage and preserving protein homeostasis in cochlear hair cells, revealing a novel mechanism involving ribosome biogenesis.

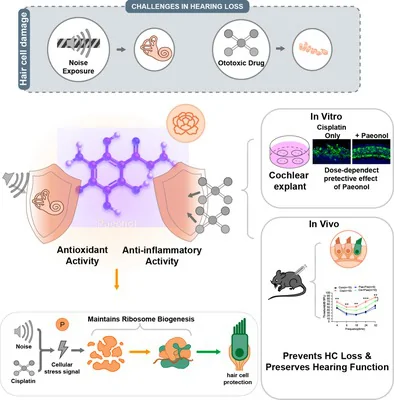

**Abbreviations**
8‐OHdG: 8‐hydroxy‐2′‐deoxyguanosine
ABR: auditory brainstem response
ANOVA: one‐way analysis of variance
ARE: antioxidant response element
Cis: cisplatin
CM‐H2DCFDA: 5‐ (and 6‐) chloromethyl‐2′,7′‐dichlorodihydrofluorescein diacetate, acetyl ester
Con: control
CoQ10: coenzyme Q10
COVID‐19: coronavirus disease 2019
DEGs: differentially expressed genes
DMEM: Dulbecco's Modified Eagle Medium
DMSO: dimethyl sulfoxide
DNA: deoxyribonucleic acid
EDTA: ethylenediaminetetraacetic acid
EGFR: epidermal growth factor receptor
FDA: Food and Drug Administration
FDR: false discovery rate
GO: Gene Ontology
GPX4: glutathione peroxidase 4
HC: hair cell
IHC: inner hair cells
IL‐6: interleukin‐6
JNK: c‐Jun N‐terminal kinase
KEGG: Kyoto Encyclopedia of Genes and Genomes
MAPK: mitogen‐activated protein kinase
NADPH: nicotinamide adenine dinucleotide phosphate
NF: neurofilaments
NF‐κB: nuclear factor kappa B
NIHL: noise induced hearing loss
Nrf2: nuclear factor erythroid 2–related factor 2
OHC: outer hair cell
P1: postnatal day 1
Pae: paeonol, 2′‐hydroxy‐4′‐methoxyacetophenone
PBS: phosphate‐buffered saline
PBST: phosphate buffered saline with Tween‐20
PCA: principal component analysis
PFA: paraformaldehyde
qPCR: quantitative real‐time polymerase chain reaction
RNA‐seq: RNA sequencing
ROS: reactive oxygen species
rRNA: ribosome RNA
SDS‐PAGE: sodium dodecyl sulfate–polyacrylamide gel electrophoresis
SEM: standard error of the mean
SNHL: sensorineural hearing loss
SPL: sound pressure level
STS: sodium thiosulfate
TBST: tris‐buffered saline with Tween‐20
TNF‐α: tumor necrosis factor‐alpha

## 1 Introduction

Sensorineural hearing loss (SNHL) is a major global health issue that severely impacts communication and quality of life [1, 2]. It can result from various factors including genetics, aging, noise, and ototoxic drugs like cisplatin (Cis) [3, 4]. Cis, a common chemotherapy agent, causes dose‐dependent hearing loss, but its clinical use is limited by ototoxicity [5, 6, 7, 8]. Although sodium thiosulfate (STS) is FDA‐approved to prevent Cis‐induced hearing loss in children, its use is limited by systemic side effects and concerns about interfering with Cis antitumor efficacy [9, 10, 11, 12]. Thus, new protective strategies are urgently needed.

Mechanisms underlying SNHL are diverse, including genetic defects, impaired autophagy, ferroptosis, inflammation, and oxidative stress. Excessive accumulation of reactive oxygen species (ROS) is considered a major contributor to acquired forms of hearing loss, including Cis‐induced, noise‐induced, and age‐related hearing loss [13]. Cis generates excessive ROS, triggering mitochondrial dysfunction and apoptosis in outer hair cells, particularly in the high‐frequency region of the cochlea [14, 15, 16, 17]. While oxidative pathways are well recognized, the precise molecular mechanisms remain unclear, highlighting the need for targeted interventions [16, 17].

Paeonol (Pae), a phenolic compound from traditional medicine [18, 19, 20], offers distinct advantages for mechanistic study due to its defined structure, low molecular weight, and favorable tissue penetration [18, 20, 21]. Known for its antioxidant and anti‐inflammatory effects, Pae protects multiple organs by activating the Nrf2 pathway and suppressing NF‐κB signaling [22, 23, 24, 25, 26, 27] pathways also implicated in Cis ototoxicity. However, whether Pae protects cochlear hair cells from Cis remains unexplored.

In the present study, we investigated the otoprotective efficacy of Pae against Cis‐induced damage using both in vitro cochlear explant cultures and in vivo models of ototoxicity. We demonstrated that Pae significantly attenuates hair cell loss, reduces ROS accumulation, and mitigates apoptosis. Notably, through transcriptomic analysis, we uncovered a previously unrecognized mechanism: Pae robustly promotes ribosomal biogenesis in auditory hair cells, a process crucial for cellular homeostasis and stress adaptation. This enhancement of ribosomal biogenesis correlates with improved hair cell survival under Cis stress. Our findings not only confirm the antioxidant role of Pae in otoprotection but also reveal a novel cytoprotective axis involving ribosomal biogenesis regulation, offering a fresh therapeutic perspective for preventing Cis‐induced hearing loss.

## 2 Materials and Methods

### 2.1 In Vitro Cochlear Epithelia Culture

Cochlear epithelia from postnatal day 1 (P1) mice were cultured on Cell‐Tek‐coated (Sigma, diluted 1:1 with phosphate‐buffered saline [PBS]) coverslips in Dulbecco's Modified Eagle Medium (DMEM)/F12 medium supplemented with N2 and B27. For injury, 30 μM cisplatin (Sigma) was added for 24 h. Explants were treated with 50, 100, or 150 μM paeonol (MCE, 10 mM stock solution in DMSO) starting 24 h before and during cisplatin exposure.

### 2.2 In Vivo Injury Model and Drug Administration

Mice received a single cisplatin injection (20 mg/kg) on day 2 [28]. The paeonol group received daily 75 mg/kg paeonol (in DMSO/corn oil) from day 1 to 3 [29]. All mice received daily saline injections. After hearing screening, mice were randomized into noise‐only or noise + paeonol groups. Noise exposure was 105 dB SPL white noise for 2 h. Paeonol was given i.p. on three consecutive days (before, during, and after noise exposure).

### 2.3 Real‐Time Polymerase Chain Reaction (qPCR)

Total RNA was extracted from cochlear epithelia using TRIzol (Invitrogen, #15596026). cDNA was synthesized using PrimeScript RT Master Mix (Takara, #RR047A). qPCR was performed in 25 μL reactions with SYBR Green Master Mix (EZB, A0012‐R2) on a QuantStudio 3 system. Primers sequences are listed in Table S1. Gene expression was normalized to β‐actin and calculated using the comparative cycle threshold (2^−ΔΔCt^) method.

### 2.4 Detection of Intracellular ROS Using CM‐H2DCFDA

ROS levels in cochlear explants were measured using CM‐H_2_DCFDA (Beyotime, S0035S). Explants were incubated with 5 μM CM‐H_2_DCFDA in serum‐free medium for 30 min at 37°C in the dark, then washed three times. Images were captured using a stereo microscope.

### 2.5 Measurement of Mitochondrial Reactive Oxygen Species (mROS)

Mitochondrial superoxide was detected using MitoSOX Red (Thermo Fisher, M36008). Cochlear explants were incubated with 5 μM MitoSOX in serum‐free medium for 30 min at 37°C under 5% CO_2_, then washed three times before imaging.

### 2.6 Auditory Brainstem Response (ABR) Test Recordings

ABR thresholds were measured using the BioSigRZ system (Tucker‐Davis Technologies, Alachua, FL, USA). Mice were anesthetized with ketamine (100 mg/kg)/xylazine (25 mg/kg) and maintained at 38°C. Thresholds were assessed at 4, 8, 16, 24, and 32 kHz using the BioSigRZ system.

### 2.7 Immunofluorescence

Cochlear explants were fixed in 4% paraformaldehyde (PFA, Sigma), washed, and blocked with 10% goat serum in 1% Phosphate Buffered Saline with Tween‐20 (PBST). Primary antibodies, including MYO7A (Proteus BioSciences, #25‐6790), 8‐OHdG (Santa Cruz, #sc‐66036), IL‐6 (R&D Systems, #AF‐406‐NA), TNF‐α (Abcam, #52B83), were applied overnight at 4°C, followed by Alexa Fluor‐conjugated secondary antibodies and DAPI (Sigma, #D9542). For in vivo tissues, cochleae were fixed, decalcified in Ethylenediaminetetraacetic Acid (EDTA), and micro‐dissected. Primary antibodies included Homer1 (Proteintech, 12433‐1‐AP), CtBP2 (BD Transduction, 612044), NF (Abcam, ab72996), Prestin (Santa Cruz Biotechnology, #sc‐22692), and Vglut3 (Synaptic Systems, 135203). Images were acquired by confocal microscopy.

### 2.8 RNA Sequencing (RNA‐Seq) and Data Analysis

To investigate Pae's protective mechanisms, RNA‐seq was performed on cochlear explants treated with cisplatin, cisplatin + 150 μM Pae, or DMSO (*n* = 3/group). Libraries were prepared using the SMART‐Seq v4 Ultra Low Input RNA Kit for Sequencing and the Illumina Stranded mRNA Prep kit (Ligation, Illumina), with size selection (300 bp) and 15‐cycle PCR amplification. After quantification using Qubit 4.0, the paired‐end RNA‐seq library was sequenced on a NovaSeq 6000 platform with a 2 × 150 bp read length. Raw Fastq files were trimmed with Trimmomatic and mapped to the mouse reference genome (mm10) using HISAT2. HTSeq was used for transcript assembly, and DESeq2 for differential expression analysis (adjusted *p* < 0.05, fold change ≥ 1.2 or ≤ 0.833). Heatmaps, Gene Ontology (GO), and Kyoto Encyclopedia of Genes and Genomes (KEGG) enrichment analyses were performed on the OECloud platform (https://cloud.oebiotech.com), with the false discovery rate (FDR) ≤ 0.05 considered significant.

### 2.9 Western Blot Analysis

Cochlear explants were dissected and basilar membranes were lysed in RIPA lysis buffer (Beyotime, P0013B). The protein samples were mixed with 5× Protein Loading Buffer (Beyotime, P0015) directly and denatured, separated by SDS‐PAGE, and transferred onto PVDF membranes (Merck, IPFL00010). The membranes were incubated overnight at 4°C with primary antibodies (Proteintech, RPLP0, Cat. No. 11290‐2‐AP; RPL10A, Cat. No. 16681‐1‐AP; RPS13, Cat. No. 16680‐1‐AP; β‐tubulin, Cat. No. 10094‐1‐AP) and then washed three times with TBST. The membranes were then incubated with HRP‐conjugated goat anti‐rabbit IgG (Beyotime, A0208) or goat anti‐mouse IgG (Beyotime, A0216) for 2 h at room temperature. Protein bands were visualized using an enhanced chemiluminescence kit (Yeasen, Cat. No. 36208ES60) and imaged using a Tanon imaging system (Tanon, Shanghai, China) after further washing with TBST three times.

### 2.10 Statistical Analysis

One‐way analysis of variance (ANOVA) followed by Tukey's multiple comparisons test was used to compare differences among groups. Data are presented as the mean ± standard error of the mean (SEM) from at least three independent assays. GraphPad Prism 6.0 (GraphPad, San Diego, CA, USA) was used for statistical analysis, *p* < 0.05 was considered statistically significant.

## 3 Results

### 3.1 Pae Attenuates Cis‐Induced Cochlear Hair Cell Loss in a Concentration‐Dependent Manner

**FIGURE 1:**
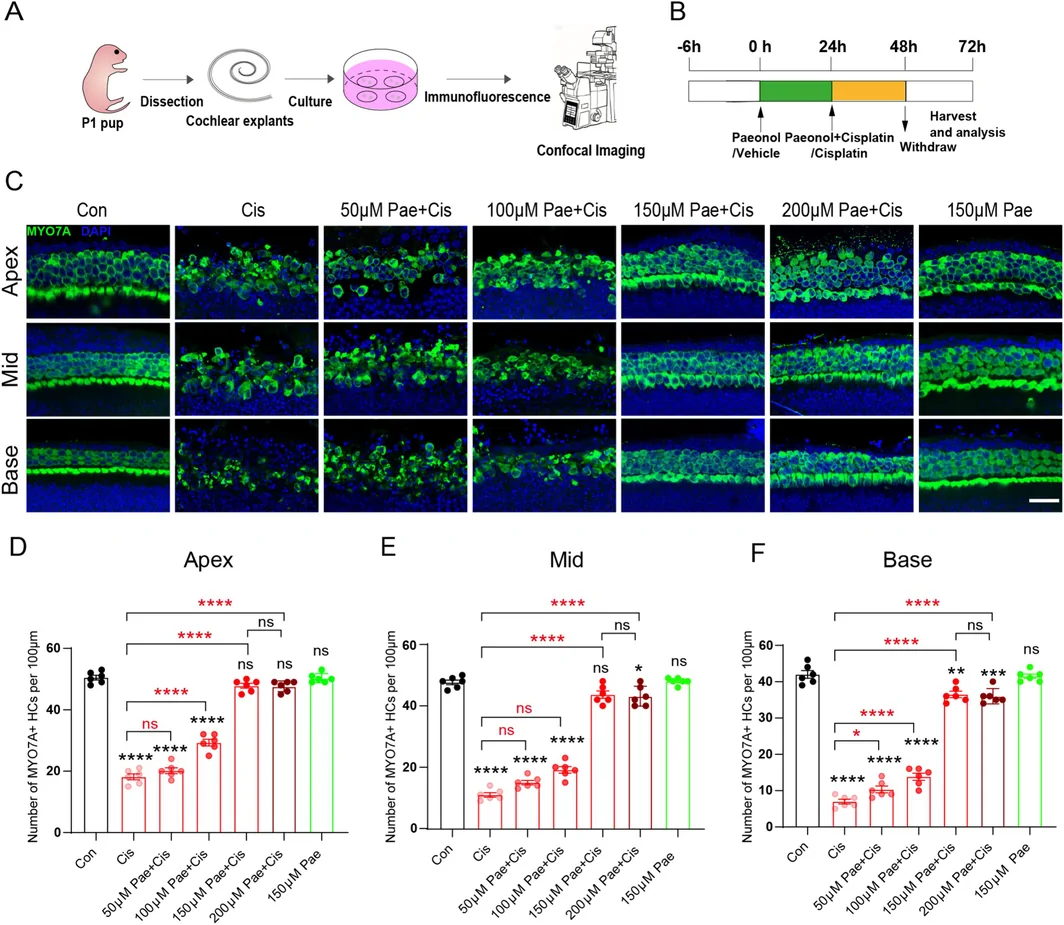
Pae confers dose‐dependent protection against Cis‐induced hair cell loss in neonatal cochlear explants. (A) Schematic overview of the experimental design. Cochlear epithelia were isolated from P1 mice and maintained in organotypic culture. (B) Treatment paradigm. Cochlear explants were allowed to adhere for 6 h, then pretreated with Pae or vehicle for 24 h, followed by Cis exposure for 24 h. After drug withdrawal, cultures were maintained for an additional 24 h before fixation for analysis (C) Representative immunofluorescence images of cochlear explants showing MYO7A‐positive hair cells (green) and DAPI‐labeled nuclei (blue) in the apical, middle and basal turns under the indicated treatment conditions. The groups include control, Cis alone, Pae (50, 100, 150 or 200 μM) combined with Cis, and Pae alone (150 μM). Scale bar: 50 μm. (D–F) Quantification of MYO7A‐positive hair cells per 100 μm in the apical (D), middle (E), and basal (F) turns under the indicated treatment conditions. Statistical significance is indicated by asterisks: Black asterisks above the bars denote comparisons of each indicated group versus Con, whereas red asterisks denote comparisons between the 50–200 μM Cis + Pae group and the Cis group. Data are presented as mean ± SEM. Statistical analysis was performed using ANOVA followed by Tukey's post hoc test. *p < 0.05, **p < 0.01, ***p < 0.001, ****p < 0.0001, ns, not significant.

To evaluate whether Pae protects cochlear hair cells from Cis‐induced injury in vitro, cochlear explants from P1 mice were pretreated with Pae (50–200 μM) for 24 h and then co‐exposed to Cis for 24 h before MYO7A immunostaining (Figure 1A–C). Pae alone did not affect hair cell survival in any cochlear turn, with comparable MYO7A^+^ hair cell densities across apical, middle, and basal regions. In contrast, Cis‐induced a marked, region‐dependent hair cell loss (*p* < 0.0001 vs. control), following a base‐to‐apex gradient. Hair cell density decreased from 50.50 ± 0.76 to 18.17 ± 0.95 cells/100 μm in the apical turn (~64% loss), from 47.67 ± 0.76 to 11.00 ± 0.73 in the middle turn (~77% loss), and from 42.00 ± 1.07 to 7.00 ± 0.63 in the basal turn (~83% loss) (Figure 1D–F).

Pae pretreatment attenuated Cis‐induced hair cell loss in a concentration‐dependent manner. In the apical turn, 100 μM, 150 μM, and 200 μM Pae increased hair cell numbers to 29.33 ± 1.09, 27.83 ± 0.79, and 47.67 ± 0.72 cells/100 μm, respectively (all *p* < 0.0001 vs. Cis), whereas 50 μM showed no significant effect (20.00 ± 0.95, *p* = 0.3776). In the middle turn, hair cell densities improved from 11.00 ± 0.73 (Cis) to 15.00 ± 0.73, 19.17 ± 1.08, 43.67 ± 1.20, and 43.17 ± 1.33 cells/100 μm with 50, 100, 150, and 200 μM Pae, respectively (all *p* < 0.001). In the basal turn, Pae increased hair cell numbers from 7.00 ± 0.63 (Cis) to 10.83 ± 0.92, 13.83 ± 0.98, 36.50 ± 0.89, and 36.00 ± 0.86 cells/100 μm (*p* < 0.005). At 150–200 μM, hair cell survival recovered to ~85%–90% of control levels.

Together, these data indicate that Pae is well tolerated and effectively mitigates Cis‐induced hair cell loss in a region‐ and concentration‐dependent manner, with 150 μM providing robust and near‐maximal protection across cochlear turns in this explant model.

### 3.2 Pae Exerts Multifaceted Protective Effects on Cochlear HCs Against Cis‐Induced Damage

**FIGURE 2:**
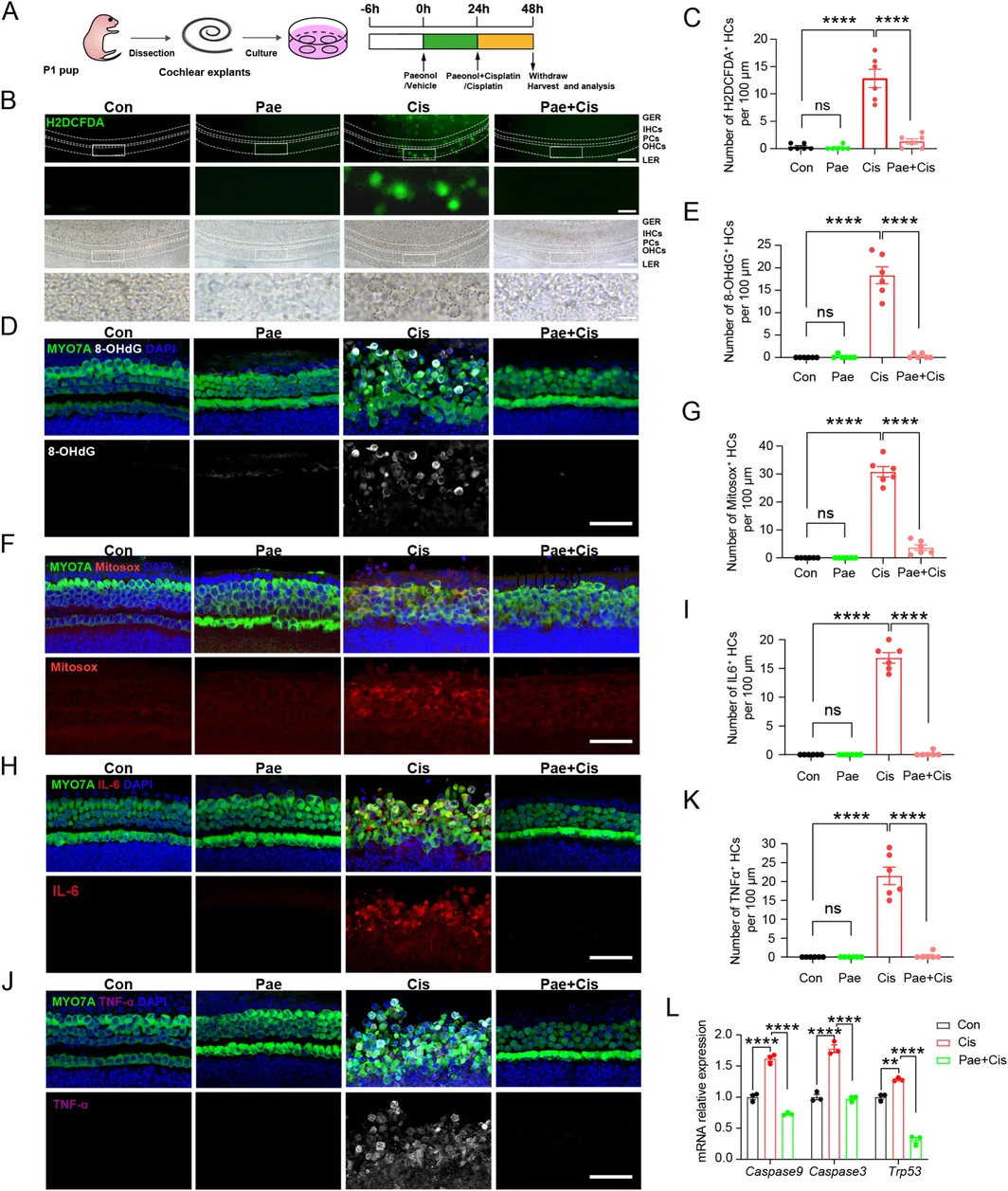
Paeonol attenuates cisplatin‐induced oxidative stress, inflammation, and apoptosis in cochlear hair cells ex vivo. (A) Schematic overview of the experimental design and treatment paradigm. Cochlear epithelia were isolated from P1 mice and maintained in organotypic culture. Cochlear explants were incubated with either Pae or vehicle for 24 h, followed by a 24 h co‐exposure period to Cis in the continued presence of the respective agent. (B) Representative images of DCFH‐DA staining (green) in the middle turn of the basilar membrane at 12 h post‐Cis exposure, showing general intracellular ROS levels. The upper two rows show fluorescence images with inset areas at inset images (25 μm) and larger fields at 100 μm. The lower two rows show corresponding MYO7A/8‐OHdG staining in the same regions. (C) Quantification of H2DCFH‐DA positive hair cells per 100 μm in the middle turn of the basilar membrane at 12 h post‐Cis exposure. (D) Representative immunofluorescence images of 8‐OHdG staining (gray) indicating oxidative DNA damage in the middle turn of the basilar membrane at 12 h post‐Cis exposure. MYO7A‐positive hair cells (green) and DAPI‐stained nuclei (blue). Scale bar, 50 μm. (E) Quantification of 8‐OHdG positive hair cells per 100 μm in the middle turn of the basilar membrane. (F) Representative immunofluorescence images of MitoSOX Red staining (red) showing mitochondrial superoxide levels in the middle turn of the basilar membrane at 12 h post‐Cis exposure. MYO7A‐positive hair cells (green) and DAPI‐stained nuclei (blue). Scale bar, 50 μm. (G) Quantification of MitoSOX red positive hair cells per 100 μm in the middle turn of the basilar membrane. (H) Representative immunofluorescence images of IL‐6 expression (red) in the middle turn of the basilar membrane at 12 h post‐Cis exposure. MYO7A‐positive hair cells (green) and DAPI‐stained nuclei (blue). Scale bar, 50 μm. (I) Quantification of IL‐6 positive hair cells per 100 μm in the middle turn of the basilar membrane. (J) Representative immunofluorescence images of TNF‐α expression (purple) in the middle turn of the basilar membrane at 12 h post‐Cis exposure. MYO7A‐positive hair cells (green) and DAPI‐stained nuclei (blue). Scale bar, 50 μm. (K) Quantification of TNF‐α positive hair cells per 100 μm fluorescence intensity in the middle turn of the basilar membrane. (L) qPCR analysis shows the relative mRNA expression levels of key apoptotic genes (*caspase‐9, caspase‐3, Trp53*) in cochlear explants after 24 h of Cis exposure. Data are presented as mean ± SEM. Statistical analysis was performed using ANOVA followed by Tukey's post hoc test. **p < 0.01, ****p < 0.0001, ns, not significant.

As 150 μM Pae provides near‐maximal protection across cochlear turns, we next examined its molecular mechanisms using P1 mouse cochlear explants treated with Control (Con), Cis, Cis with Pae pretreatment (Cis + Pae), and Pae alone (Pae) (Figure 2A). As excessive ROS accumulation and lipid peroxidation are central drivers of ototoxic hair cell death [30, 31, 32], we first assessed oxidative stress (Figure 2B–G). Pae pretreatment markedly attenuated Cis‐induced oxidative stress, significantly the number of ROS‐positive HCs (1.33 ± 0.49 vs. 12.83 ± 1.66, *p* < 0.0001), 8‐OHdG positive HCs (0.33 ± 0.21 vs. 18.33 ± 1.89, *p* < 0.0001), and MitoSOX red‐positive HCs (3.67 ± 0.99 vs. 30.83 ± 1.85, *p* < 0.0001).

In addition to oxidative stress, inflammation contributes significantly to Cis‐induced ototoxicity [33, 34]. We examined the expression of pro‐inflammatory cytokines IL‐6 and TNF‐α in the cochlear explants (Figure 2H–K). Cis treatment markedly increased the number of IL‐6‐positive HCs (16.83 ± 0.91 vs. 0.00 ± 0.00, *p* < 0.0001 vs. Con) and TNF‐α positive HCs similarly elevated (21.50 ± 2.34 vs. 0.00 ± 0.00, *p* < 0.0001 vs. Con). Pae treatment significantly suppressed these increases (IL‐6: 0.17 ± 0.17; TNF‐α: 0.33 ± 0.33; both *p* < 0.0001 vs. Cis).

Given that Cis triggers intrinsic apoptosis, we next evaluated the expression of key apoptotic markers, Trp53, caspase‐9, and caspase‐3, to examine the protective effects of Pae [35, 36, 37]. Cis treatment significantly upregulated Trp53 (1.03 ± 0.01 vs. 1.00 ± 0.02, *p* < 0.0001), caspase‐9 (1.62 ± 0.04 vs. 1.00 ± 0.04, *p* < 0.0001), and caspase‐3 (1.78 ± 0.06 vs. 1.00 ± 0.04, *p* < 0.0001). Pae pretreatment markedly suppressed these pro‐apoptotic changes, reducing Trp53 to 0.86 ± 0.02, caspase‐9 to 0.73 ± 0.01, and caspase‐3 to 0.97 ± 0.03 (all *p* < 0.0001 vs. Cis), indicating effective inhibition of Cis‐induced intrinsic apoptotic activation (Figure 2L).

Collectively, these results indicate that Pae exerts robust protective effects against Cis‐induced cochlear injury by suppressing mitochondrial oxidative stress, attenuating inflammatory responses, and inhibiting the activation of intrinsic apoptotic pathways, while Pae alone exhibited no detectable adverse effects.

### 3.3 Pae Protects Hearing and Preserves Synapse Integrity in Cis‐Treated Mice

**FIGURE 3:**
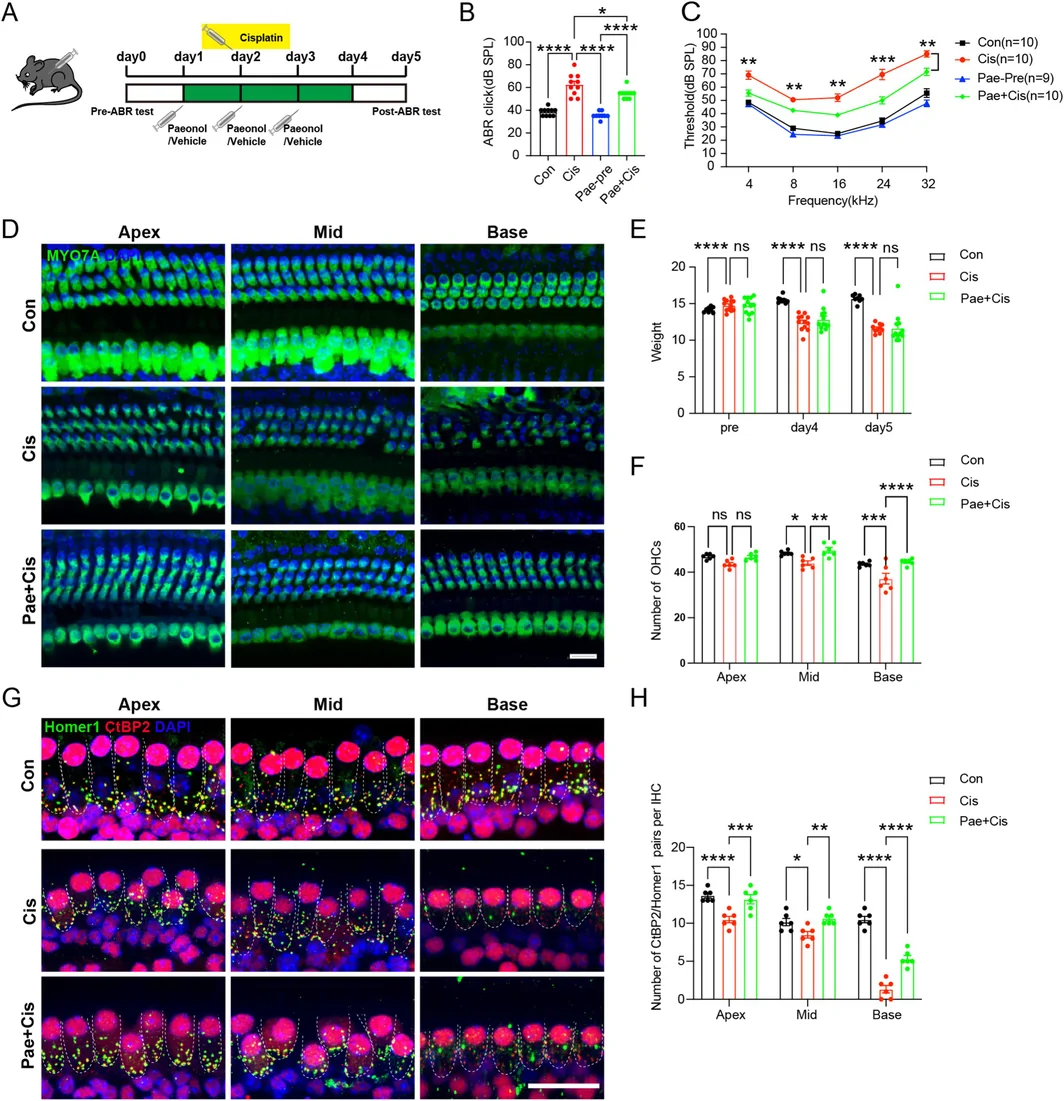
Pae attenuates Cis‐induced auditory threshold shifts and preserves ribbon synapses in cochlear inner hair cells in vivo. (A) Schematic diagram illustrating the experimental timeline. Baseline ABR testing was performed on day 0. Pae or vehicle was administered once daily from day 1 to day 3. Cis was administered on day 2. Post‐treatment ABR testing was conducted on day 5. (B) ABR thresholds measured using click stimuli, expressed in decibels sound pressure level (dB SPL). *n* = 10 mice per group (Pae‐Pre, *n* = 9). (C) ABR thresholds measured using tone‐burst stimuli at frequencies of 4, 8, 16, 24, and 32 kHz. Thresholds are expressed in decibels sound pressure level (dB SPL). *n* = 10 mice per group, Pae‐Pre, *n* = 9. (D) Representative confocal images of IHCs and OHCs in the apical, middle, and basal turns of the cochlea. Hair cells were labeled with MYO7A (green), and nuclei were counterstained with DAPI (blue). Scale bar: 50 μm. (E) Body weight changes in the three groups at pretreatment and on days 4 and 5 after Cis administration. (F) Quantification of MYO7A‐positive OHCs per 100 μm in the apical, middle, and basal turns of the cochlea under conditions of acute injury. (G) Representative confocal images of IHC regions from the apical, middle, and basal turns of the cochlea. Presynaptic ribbons were labeled with CtBP2 (red), postsynaptic densities with Homer1 (green), and nuclei with DAPI (blue). Scale bar: 50 μm. (H) Quantification of juxtaposed CtBP2/Homer1 synaptic pairs per IHC in the apical, middle, and basal turns of the cochlea. Data are presented as mean ± SEM. Statistical analysis was performed using ANOVA followed by Tukey's post hoc test. *p < 0.05, **p < 0.01, ***p < 0.001, ****p < 0.0001, ns, not significant.

We further investigated the protective effect of Pae against Cis‐induced ototoxicity in vivo (Figure 3A). ABR testing revealed that Cis markedly elevated click‐evoked thresholds compared with controls (62.50 ± 3.01 dB vs. 38.50 ± 1.07 dB, *p* < 0.0001), confirming cochlear dysfunction. Pae significantly attenuated this elevation (54.50 ± 1.38 dB, *p* = 0.0235 vs. Cis) without affecting baseline thresholds (Figure 3B). Pure‐tone ABR revealed frequency‐dependent threshold shifts after Cis exposure, with pronounced increases at 8 kHz (50.50 ± 1.74 dB vs. 29.00 ± 1.00 dB), 16 kHz (52.00 ± 2.91 dB vs. 25.00 ± 1.67 dB), 24 kHz (69.50 ± 3.91 dB vs. 34.50 ± 2.41 dB), and 32 kHz (85.00 ± 2.36 dB vs. 55.50 ± 3.29 dB; all *p* < 0.0001). Pae significantly reduced threshold shifts at these frequencies (8 kHz, 42.50 ± 1.54 dB; 16 kHz, 39.00 ± 1.94 dB; 24 kHz, 50.00 ± 2.69 dB; 32 kHz, 71.50 ± 2.79 dB; all *p* < 0.001), with a partial, non‐significant improvement at 4 kHz (*p* = 0.078) (Figure 3C).

Baseline body weights were similar across groups (Con 14.15 ± 0.13 g; Cis 14.75 ± 0.22 g; Pae + Cis 14.73 ± 0.31 g, ns), indicating comparable initial conditions (Figure 3E). Cis administration caused marked weight loss on days 4 and 5 compared with controls (day 4 12.42 ± 0.31 g vs. 15.50 ± 0.17 g; day 5 11.60 ± 0.19 g vs. 15.68 ± 0.19 g; both *p* < 0.0001). Pae pretreatment had no significant effect on body weight (day 4 12.84 ± 0.45 g; day 5 11.64 ± 0.59 g, *p* = 0.8535). Importantly, survival was significantly improved, with six deaths in the Cis group compared with two in the Pae + Cis group and none in controls.

Cochlear hair cell analysis showed segment‐specific damage by Cis and protection by Pae (Figure 3D,F). Cis caused significant OHC loss in the middle turn (44.00 ± 0.97 vs. 48.33 ± 0.42, *p* = 0.0138) and basal turns (37.17 ± 2.36 vs. 43.50 ± 0.43, *p* < 0.001), with a non‐significant trend at the apical turn. Pae pretreatment restored OHC counts toward control levels, significantly improving middle turn (49.67 ± 1.17, *p* = 0.0011 vs. Cis) and basal turn counts (*p* < 0.001). IHCs were largely preserved, with middle‐turn IHCs partially protected by Pae (*p* = 0.006 vs. Cis; *p* = 0.680 vs. Con), and basal IHCs unchanged. Cis also markedly reduced IHC ribbon synapse numbers throughout the cochlea, with synapse counts decreasing from 13.67 ± 0.00 to 10.50 ± 0.00 in the apical turn (*p* = 0.0007), from 10.17 ± 0.00 to 8.50 ± 0.00 in the middle turn (*p* = 0.0324), and from 10.50 ± 0.00 to 1.33 ± 0.00 in the basal turn (*p* < 0.0001), indicating a clear tonotopic pattern of synaptopathy (Figure 3G,H). Pae treatment significantly preserved ribbon synapses in the apical (13.17 ± 0.00, *p* = 0.0029 vs. Cis) and middle turn (10.67 ± 0.00, *p* = 0.0060 vs. Cis), restoring synapse numbers to near or above control levels, and partially rescued synaptic loss in the basal turn (5.33 ± 0.00, *p* < 0.0001 vs. Cis).

These data demonstrate that Pae preserves cochlear integrity and auditory function under Cis‐induced stress by attenuating hair cell loss and maintaining ribbon synapse integrity.

### 3.4 Pae Mitigates Noise‐Induced Hearing Loss and Preserves Cochlear Synapses

**FIGURE 4:**
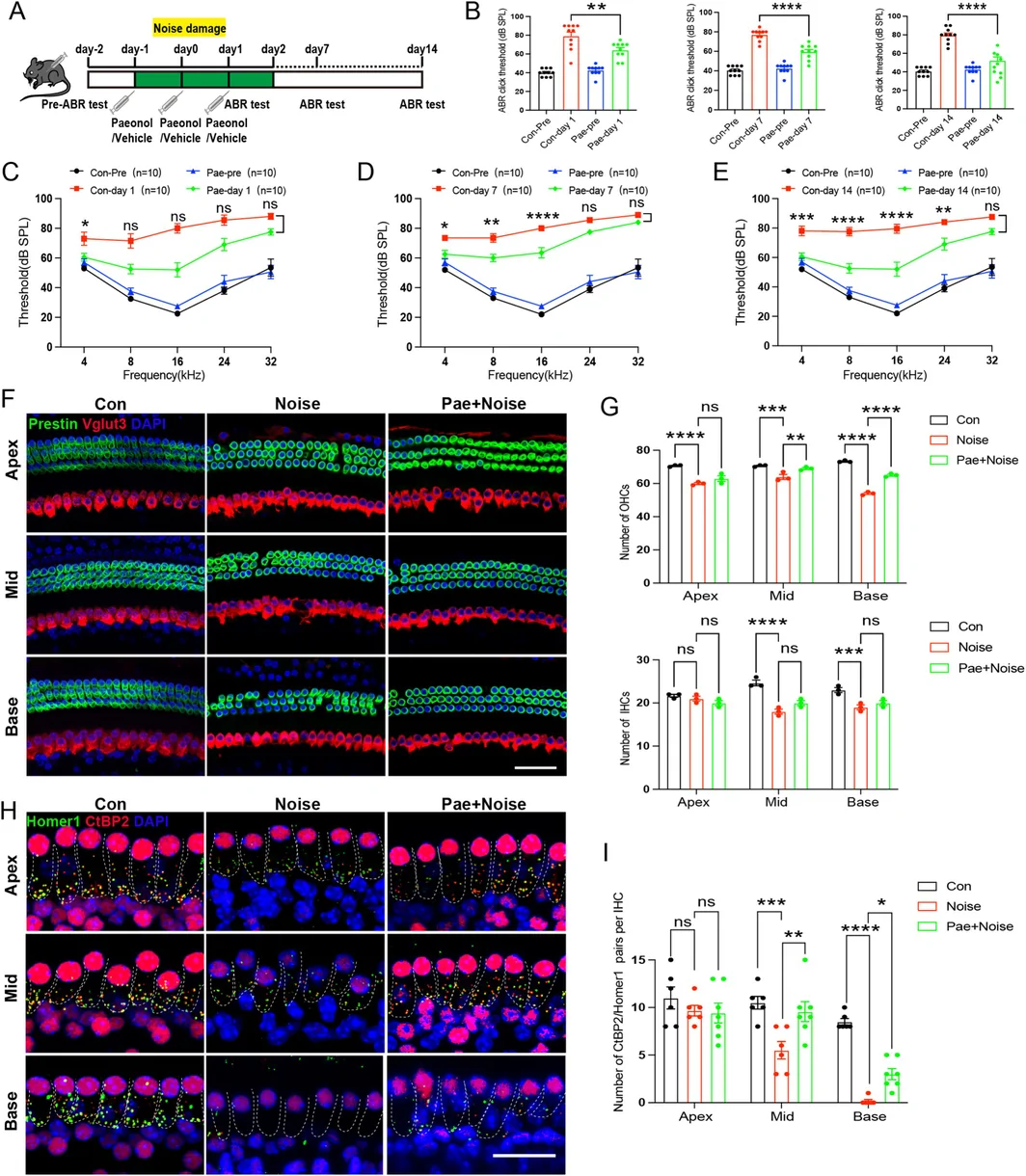
Pae protects against noise‐induced hearing loss and cochlear hair cell damage. (A) Schematic illustration of the experimental timeline. Mice received Pae or vehicle administration before and after noise exposure on day 0. ABR thresholds were measured before noise exposure and at days 1, 7, and 14 after noise exposure. (B) ABR click thresholds measured before and at 1, 7, and 14 days post‐noise exposure in control and Pae‐treated mice (*n* = 10 per group). (C–E) ABR threshold shifts across frequencies from 4 to 32 kHz measured at day 1 (C), day 7 (D), and day 14 (E) after noise exposure (*n* = 10 per group). (F) Representative whole‐mount cochlear immunofluorescence images showing outer hair cells labeled with Prestin (green), inner hair cells labeled with Vglut3 (red), and nuclei counterstained with DAPI (blue) in the apical, middle, and basal turns of the cochlea from noise‐exposed mice treated with vehicle or Pae. (G) Quantification of outer hair cells (upper panel) and inner hair cells (lower panel) in the apical, middle, and basal turns of the cochlea. (H) Representative whole‐mount images of inner hair cell ribbon synapses stained for CtBP2 (red) and Homer1 (green) in the apical, middle, and basal turns of the cochlea from control, noise‐exposed, and Pae‐treated mice. (I) Quantification of juxtaposed CtBP2/Homer1 synaptic pairs per IHC in the apical, middle, and basal turns of the cochlea. Data are presented as mean ± SEM. Statistical analysis was performed using ANOVA followed by Tukey's post hoc test. *p < 0.05, **p < 0.01, ***p < 0.001, ****p < 0.0001, ns, not significant.

To assess the protective effect of Pae against NIHL, auditory function was evaluated by click‐evoked and pure‐tone ABR at 1, 7, and 14 days after noise exposure (Figure 4A). One day after noise exposure, click‐evoked ABR thresholds were markedly elevated in vehicle‐treated mice compared with baseline values, confirming successful induction of NIHL (79.00 ± 4.33 dB vs. 40.00 ± 1.29 dB, *p* < 0.0001). Pae‐treated mice exhibited significantly smaller threshold shifts at this acute stage, with thresholds reduced to 64.00 ± 2.77 dB (*p* = 0.0028 vs. Noise), indicating partial preservation of auditory function immediately after acoustic trauma. At 7 days post‐exposure, thresholds in the vehicle group remained severely elevated (77.00 ± 1.86 dB), whereas Pae treatment promoted significant functional recovery, lowering thresholds to 59.50 ± 2.41 dB (*p* < 0.0001 vs. Noise). This protective effect persisted through 14 days, as Pae‐treated mice maintained substantially lower click‐evoked thresholds (52.00 ± 4.10 dB) compared with vehicle‐treated controls (79.50 ± 2.52 dB, *p* < 0.0001), demonstrating sustained auditory protection (Figure 4B).

Pure‐tone ABR analysis revealed frequency‐dependent threshold elevations after noise exposure, particularly at mid‐to‐high frequencies (16 kHz: 80.00 ± 3.25 dB; 32 kHz: 88.00 ± 2.00 dB), which were significantly attenuated by Pae at most frequencies (4 kHz: 60.50 ± 2.63 dB, *p* = 0.0285; 8 kHz: 52.50 ± 3.18 dB, *p* = 0.0004; 16 kHz: 52.00 ± 4.78 dB, *p* < 0.0010; 24 kHz: 69.00 ± 4.14 dB, *p* = 0.0023), with partial, non‐significant improvement at 32 kHz (77.50 ± 2.14 dB, *p* = 0.0804). By day 7, Pae‐treated mice showed greater recovery at low‐to‐mid frequencies (4 kHz: 60.50 ± 2.61 dB vs. 73.50 ± 1.30 dB, *p* = 0.0125; 8 kHz: 60.00 ± 2.58 dB vs. 73.50 ± 2.99 dB, *p* = 0.0015; 16 kHz: 63.50 ± 3.50 dB vs. 80.00 ± 1.05 dB, *p* < 0.0010), with limited improvement at higher frequencies. Protective effects persisted at day 14 across 4–24 kHz (4 kHz: *p* = 0.0003; 8 kHz: *p* < 0.0010; 16 kHz: *p* < 0.0010; 24 kHz: *p* = 0.0025), with a strong trend at 32 kHz (*p* = 0.0662) (Figure 4C–E).

Cochlear hair cell analysis revealed milddle OHC loss following noise exposure, with the greatest reduction observed in the basal turn (Figure 4F,G). OHC counts decreased from 70.67 ± 0.33, 70.67 ± 0.33, and 73.33 ± 0.33 in Con to 60.00 ± 0.58, 64.00 ± 1.53, and 54.00 ± 0.58 in the apical, middle, and basal turns, respectively. Pae treatment attenuated OHC loss, increasing OHC counts to 63.00 ± 1.73, 69.00 ± 0.58, and 65.00 ± 0.58 in the corresponding cochlear turns, with the greatest protection observed in the basal turn (*p* < 0.0001). In contrast, IHCs were largely preserved following noise exposure. Synapse counts exhibited a tonotopic reduction, decreasing from 11.00 ± 1.16 to 9.67 ± 0.56 in the apical turn (*p* = 0.4965), from 10.50 ± 0.67 to 5.50 ± 0.92 in the middle turn (*p* = 0.0003), and from 8.50 ± 0.34 to 0.17 ± 0.17 in the basal turn (*p* < 0.0001). Pae significantly rescued synapse loss in the middle (9.57 ± 1.04, *p* = 0.0021) and basal turns (3.00 ± 0.58, *p* = 0.0407), whereas no significant effect was observed in the apical turn (9.43 ± 1.04, *p* = 0.9758) (Figure 4H,I).

These results indicate that Pae predominantly protects cochlear synapses rather than hair cells and confers robust protection against NIHL, consistent with the tonotopic ABR recovery pattern.

### 3.5 Pae Modulates Cis‐Induced Transcriptomic Dysregulation by Restoring Ribosome Related Homeostasis and Synaptic Integrity

**FIGURE 5:**
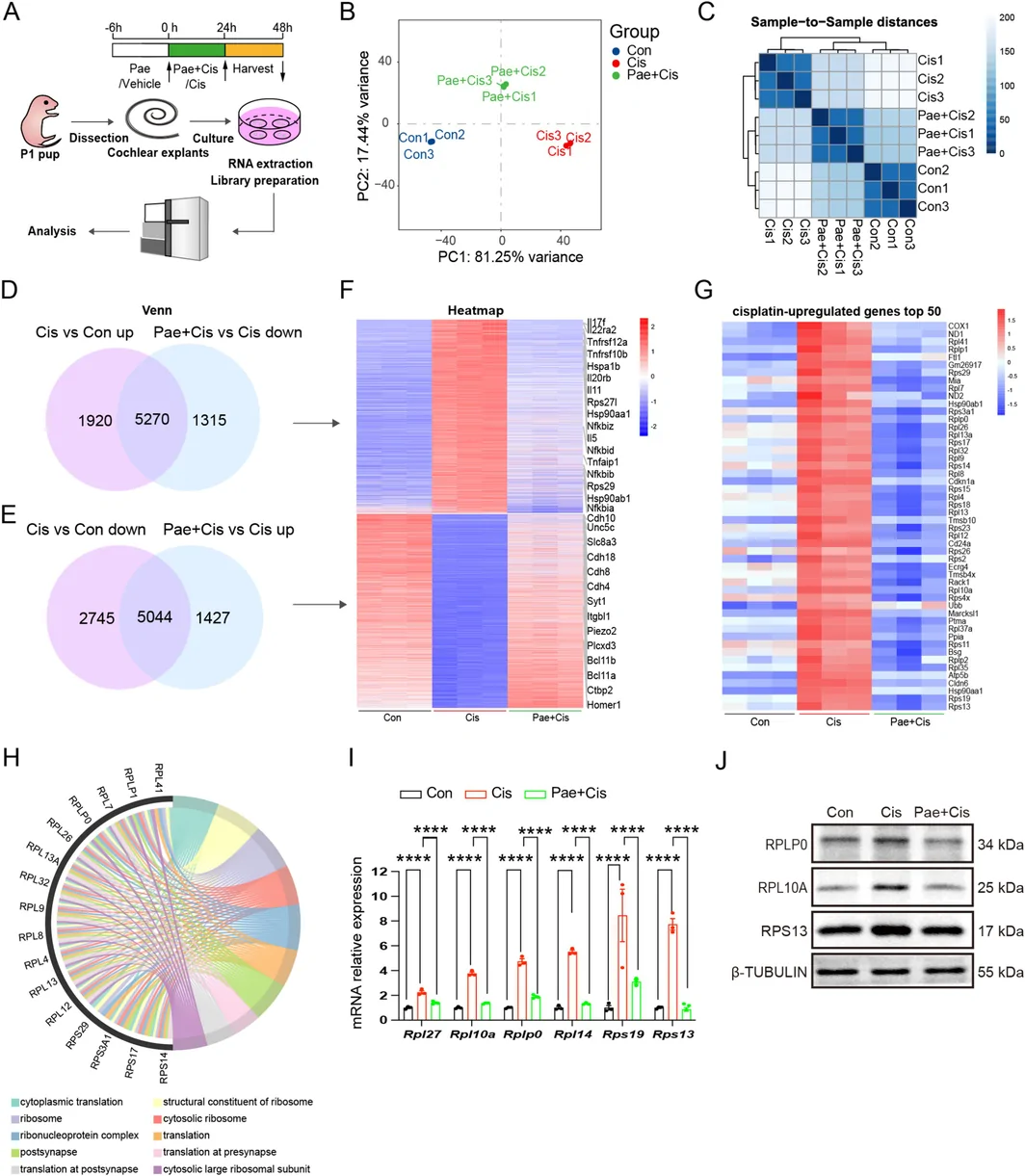
Pae restores transcriptional homeostasis by modulating ribosome biogenesis and synaptic integrity pathways altered by Cis. (A) Schematic of the experimental timeline. Cochlear explants from postnatal day 1 (P1) mice were acclimatized for 6 h, pretreated with Pae or Con from 0 h, exposed to Cis at 24 h, and harvested at 48 h for RNA sequencing. Three groups were established: Con, Cis, and Pae + Cis. (B) PCA of transcriptomic profiles. Samples from Con, Cis, and Cis + Pae separate clearly, with tight clustering of replicates within each group. (C) Sample‐to‐sample distance heatmap showing relationships among samples. Cis samples cluster separately, while Pae + Cis samples are positioned closer to Con, indicating partial transcriptional rescue. (D, E) Venn diagrams of genes consistently regulated by Cis and reversed by Pae. (D) Genes upregulated by Cis and downregulated by Cis + Pae. (E) Genes downregulated by Cis and restored by Cis + Pae. (F) Heatmap of selected DEGs. Cis upregulates inflammatory/NF‐κB‐related genes and downregulates synaptic/adhesion‐related genes. (G) Heatmap of the top 50 Cis‐upregulated genes modulated by Pae. (H) GO chord plot of the “Cis up and Pae down” gene set, showing associated biological functions. (I) qPCR analysis of six representative ribosome‐associated genes identified by RNA sequencing, including *Rpl27, Rpl10a, Rplp0, Rpl14, Rps19, and Rps13*. (J) Western blot analysis of RPLP0, RPL10A, and RPS13 protein expression in cochlear explants following the indicated treatments. Data are presented as mean ± SEM. Statistical analysis was performed using ANOVA followed by Tukey's post hoc test. ****p < 0.0001, ns, not significant.

To elucidate the protective mechanisms of Pae against Cis‐induced ototoxicity, we performed transcriptomic profiling of cochlear tissues under different treatment conditions (Figure 5A). Principal component analysis (PCA) demonstrated clear separation among control, Cis, and Pae + Cis groups (Figure 5B). Sample‐to‐sample distance analysis confirmed these differences and indicated that Pae partially restored the Cis‐induced transcriptomic profile toward that of controls (Figure 5C). Differential expression analysis revealed extensive transcriptomic alterations following Cis exposure (fold change ≥ 1.2 or ≤ 0.833, *p* < 0.05). Notably, comparison of Pae + Cis versus Cis identified thousands of genes oppositely regulated by Pae. Venn analysis showed that 5270 Cis‐upregulated genes and 5044 Cis‐downregulated genes were reversed or restored by Pae (Figure 5D,E).

Functional enrichment analysis revealed that upregulated genes in Cis were mainly involved in inflammatory response and NF‐κB signaling, while downregulated genes were mainly involved in synaptic function, cytoskeletal organization and neural structural pathways. Critically, Pae partially rescued these transcriptional disruptions (Figure 5F). Cis downregulated and Pae restored the top 50 genes were analyzed by heatmap, which indicated that Pae partially rescued the Cis inhibited genes involved in synaptic and structural functions (Figure S1A), consistent with this observation. GO enrichment analysis of this gene set revealed significant enrichment of pathways related to extracellular matrix organization, collagen and sensory perception of sound, which are consistent with the protective effects of Pae on cochlear structural integrity and auditory function (Figure S1B). Next, we identified the key pathways underlying Pae protection by focusing on the top 50 genes reversed by Pae. Ribosome‐related processes such as cytoplasmic translation and structural constituent of ribosome were significantly enriched in cis‐upregulated and Pae‐suppressed genes (Figure 5G,H). To further validate the transcriptomic findings, we selected six ribosome biogenesis‐ and ribosomal protein‐related genes with relatively high basal expression and significant differential expression in the RNA‐seq dataset, including Rpl27, Rpl10a, Rplp0, Rpl14, Rps19, and Rps13 (Figure 5I). We further selected three representative ribosome‐related genes (Rpl10a, Rplp0, and Rps13) for protein validation by Western blot analysis. Consistent with the transcriptomic and qPCR findings, Cis treatment significantly increased the protein expression of RPL10A, RPLP0, and RPS13, whereas Pae administration markedly attenuated these elevations in cochlear explants (Figure 5J). Together, these findings identify ribosome and protein synthesis machinery as a key downstream axis modulated by Pae.

## 4 Discussion

SNHL is a highly prevalent sensory disorder with limited effective clinical interventions. In this study, Pae protected against Cis‐ and noise‐induced hearing loss by modulating oxidative stress, preserving hair cell and synaptic integrity, and highlighting its potential as a candidate antioxidant‐based therapeutic strategy.

Oxidative stress and mitochondrial dysfunction are central mechanisms in acquired SNHL, acting as common downstream events after ototoxic drug or noise exposure. Excessive ROS disrupt cochlear cellular homeostasis, leading to progressive hair cell loss, synaptic degeneration, and elevated hearing thresholds [38, 39, 40, 41]. Antioxidant therapies have long been explored to mitigate cochlear damage. Classical antioxidants such as amifostine, Ebselen, and mitochondrial antioxidants like Coenzyme Q10 (CoQ10) show variable protection in Cis‐ or noise‐induced ototoxicity models [42, 43, 44, 45], supporting the translational potential of redox modulation. Recent studies have identified small extracellular vesicles (sEVs) as novel mediators of cisplatin‐induced cochlear injury, providing additional insights into the molecular mechanisms underlying acquired hearing loss [46]. Previous studies have demonstrated that oxidative stress‐targeted therapies for NIHL can be enhanced through the use of novel biomaterials including N‐acetylcysteine‐loaded silk microcarriers, mesoporous silica nanoparticles (MMSNs), *Lycium barbarum* glycopeptide (LbGP), and the NPR36–Spirulina platensis complex [47, 48, 49]. However, clinical translation remains limited due to variable efficacy and safety concerns. In contrast, systemic administration of Pae in our models provided significant protection without affecting body weight or baseline hearing, demonstrating superior safety and controllability. This advantage highlights Pae's potential for translational application. Nevertheless, we did not measure plasma or cochlear pharmacokinetics, which should be addressed in future studies [22]. Additionally, the blood‐labyrinth barrier may impede drug delivery to the inner ear following systemic administration. Further studies are needed on new delivery strategies such as intratympanic administration and nanoparticle‐based carriers that may improve the availability of Pae in the cochlea. The present study focused on acute Cis‐ and noise‐exposure models. Whether the protective effects of Pae can be maintained under chronic or repeated exposure conditions remains to be determined.

Cis‐induced ototoxicity involves not only oxidative damage but also nicotinamide adenine dinucleotide phosphate (NADPH) oxidase activation, inflammatory cascades, transient receptor potential channels, and apoptotic signaling [17, 50, 51, 52, 53]. Consistent with the complex pathogenesis of acquired SNHL, recent studies have further identified Cidea‐mediated hair‐cell apoptosis and XIAP‐DDRGK1‐dependent ER‐phagy as critical regulators of cochlear hair‐cell survival, highlighting novel therapeutic targets for both ototoxic drug‐ and noise‐induced hearing loss [54, 55]. Ferroptosis‐related factors such as glutathione peroxidase 4 (GPX4) and lipid peroxidation also play critical roles in cochlear pathology [17, 50, 56, 57]. Specifically, GPX4 rather than FSP1 is indispensable for cochlear hair cell maintenance, and pharmacological activation of GPX4 by selenomethionine or reduction of intracellular ferrous ions by luteolin effectively attenuates cisplatin‐induced ferroptotic hair cell death [56, 58]. Gasdermin D‐mediated pyroptosis in supporting cells has been identified as a key driver of noise‐induced cochlear injury, operating through an EGFR/ERK–caspase‐11‐dependent positive feedback loop between supporting cells and hair cells [59]. Our RNA‐seq data revealed significant disruption of protein homeostasis and ribosome‐related pathways [60, 61], suggesting that ototoxic stress may trigger broader translational dysregulation barely addressed in prior studies [62]. This finding indicates that Pae may exert protection by maintaining protein homeostasis and ribosome function, providing novel mechanistic insights. qPCR and Western blot validation supported the differential expression of several ribosome‐related genes. However, ribosomal activity and translational function were not directly assessed in the present study. Further studies are required to determine whether the restoration of ribosomal function is mechanistically required for the otoprotective effects of Pae. However, no significant changes in the expression of these ribosome‐related genes were observed in the in vivo noise‐induced hearing loss model following Pae treatment (Figure S2C). This finding suggests that the modulation of ribosome‐related pathways by Pae may depend on the underlying mechanism of cochlear injury and may therefore be more relevant to cisplatin‐induced ototoxicity than to noise‐induced hearing loss.

Recent work indicates that ribosome homeostasis not only involves its biogenesis and assembly, but also integrates stress signaling pathways with profound effects on cell fate decisions such as apoptosis and aging under adverse conditions [63, 64]. In addition, oxidative stress has been shown to directly impact ribosomal components or translational mechanisms, whereby chemical modifications of ribosome RNA (rRNA) and ribosomal proteins under ROS challenge diminish translational fidelity and disrupt protein synthesis, contributing to proteostasis imbalance [65, 66]. Persistent impairment of ribosome function may further activate stress‐response pathways, reduce the synthesis of cytoprotective proteins, and ultimately facilitate apoptosis under sustained oxidative stress. Consistent with our RNA‐seq findings, oxidative stress in the cochlea may similarly perturb ribosome‐associated pathways, leading to disrupted protein homeostasis and heightened hair cell vulnerability, suggesting ribosome dysregulation as a downstream consequence of oxidative injury.

## 5 Conclusions

In summary, our study reveals the multi‐layered protective mechanism of Pae: it mitigates oxidative damage and preserves protein homeostasis pathways, distinguishing it from other antioxidant candidates. Our findings demonstrate the safety of systemic Pae administration, preservation of hair cells and synapses, and identification of a novel mechanism through RNA‐seq, providing strong support for its translational potential. Future studies should clarify Pae pharmacokinetics in systemic circulation and the cochlea, assess alternative delivery methods such as intratympanic injection or sustained‐release formulations, and explore combination strategies with other ferroptosis‐ or apoptosis‐targeted otoprotective agents.

## Author Contributions

Mingyu Xia, Jiaoyao Ma, Hui Chai, and Hua Jiang designed the study. Minhui Xia, Jiaoyao Ma, Wenyu Hou, Tianshi Sun, Yiyun Lou, and Yunjie Li performed the experiments and analyzed the data. Wenyu Hou, Jiaoyao Ma, and Tianshi Sun contributed to writing the manuscript with participation from other authors. Mingyu Xia, Hua Jiang, and Jiaoyao Ma provided funding. All authors read and approved the final manuscript.

## Funding

This research was funded by National Natural Science Foundation of China (grant number 8247118 and 82501411), Shanghai “Rising Stars of Medical Talents” Youth Development Program (No. SHWSRS (2025) 071), and the grant of the Key R and D Program of Zhejiang (2024C03238).

## Ethics Statement

The animal study protocol was approved by the Ethics Committee of the Department of Laboratory Animal Science at Fudan University (protocol code 202107010S and approval date July 22, 2021).

## Conflicts of Interest

The authors declare no conflicts of interest.

## Supporting information


**Figure S1:** Paeonol restores cisplatin‐suppressed synaptic and structural gene networks.

**Figure S2:** Paeonol suppresses cisplatin‐induced apoptosis but does not alter basal ribosomal gene expression after noise exposure.

**Table S1:** Primers used for qPCR in this study.

## Data Availability

The data that support the findings of this study are openly available in Sequence Read Archive (SRA) at https://www.ncbi.nlm.nih.gov/sra/PRJNA1381264, reference number PRJNA1381264.
