# Supplementary material for: Paeonol Alleviate Cisplatin‐ and Noise‐Induced Hearing Loss by Preserving Hair Cell Ribosome Biogenesis
Source: CNS Neurosci Ther. 2026 Sep 3;32(9):e71115. doi: 10.1002/cns.71115 (PMC13539212; doi:10.1002/cns.71115)
Supplement: Supplementary file 1 [file CNS-32-e71115-s001.docx]

**Figure S1. Paeonol restores cisplatin-suppressed synaptic and structural gene** **networks.**

(A) Heatmap of the top 50 cisplatin-downregulated genes modulated by paeonol.

(B) GO chord plot of the “cisplatin-down and paeonol-up” gene set.
