# Supplementary material for: Paeonol Alleviate Cisplatin‐ and Noise‐Induced Hearing Loss by Preserving Hair Cell Ribosome Biogenesis
Source: CNS Neurosci Ther. 2026 Sep 3;32(9):e71115. doi: 10.1002/cns.71115 (PMC13539212; doi:10.1002/cns.71115)
Supplement: Supplementary file 2 [file CNS-32-e71115-s002.docx]

| Primers | Forward sequence (5’-3’) | Reverse sequence (5’-3’) |
| --- | --- | --- |
| *Caspase 3* | TGGTGATGAAGGGGTCATTTATG | TTCGGCTTTCCAGTCAGACTC |
| *Caspase 9* | TCCTGGTACATCGAGACCTTG | AAGTCCCTTTCGCAGAAACAG |
| *Trp53* | CTCTCCCCCGCAAAAGAAAAA | CGGAACATCTCGAAGCGTTTA |
| *β-actin* | AGAGGGAAATCGGTGAC | CAATAGTGATGACCTGGCCGT |
| *Rpl27* | AAAGCCGTCATCGTGAAGAAC | GCTGTCACTTTCCGGGGATAG |
| *Rpl10a* | TGGACATCGAGGCGCTCAAGAA | GAACTTGCCAGCCTTGTTTAGGC |
| *Rplp0* | AGATTCGGGATATGCTGTTGGC | TCGGGTCCTAGACCAGTGTTC |
| *Rpl14* | GGGTGGCCTACATTTCCTTCG | CTTGGCCCATCTTGTGGCT |
| *Rps19* | CAGCAGGAGTTCGTCAGAGC | CACCCATTCGGGGACTTTCA |
| *Rpsl3* | TCCCTCCCAGATAGGTGTAATCC | TCCTTTCTGTTCCTCTCAAGGT |

Table S1. Primers used for qPCR in this study.
