# Supplementary material for: Paeonol Alleviate Cisplatin‐ and Noise‐Induced Hearing Loss by Preserving Hair Cell Ribosome Biogenesis
Source: CNS Neurosci Ther. 2026 Sep 3;32(9):e71115. doi: 10.1002/cns.71115 (PMC13539212; doi:10.1002/cns.71115)
Supplement: Supplementary file 3 [file CNS-32-e71115-s003.docx]

**Figure S2. Paeonol suppresses cisplatin-induced apoptosis but does not alter basal ribosomal gene expression after noise exposure.**

(A) Representative images of TUNEL staining in cochlear explants from the Con, Cis, and Pae+Cis groups. Hair cells were labeled with MYO7A (green), apoptotic cells with TUNEL (red), and nuclei with DAPI (blue). Scale bar, 50 µm.

(B) Quantification of TUNEL-positive hair cells per 100 µm in the three groups. Paeonol pretreatment significantly reduced cisplatin-induced hair cell apoptosis.

(C) Quantitative PCR analysis of representative ribosome-associated genes (*Rpl27*, *Rpl10a*, *Rplp0*, *Rpl14*, *Rps19*, and *Rpl3*) in cochlear explants from the Con, Noise, and Noise+Pae groups.

Data are presented as mean ± SEM. Statistical analysis was performed using one-way ANOVA followed by Tukey’s post hoc test. *****P* < 0.0001, ns, not significant.
